# Super-Oxygenation as a Therapeutic Measure, with Notes of Cases Treated

**Published:** 1869-02-15

**Authors:** S. S. Wallihan

**Affiliations:** Chicago


### *Super-Oxygenation as a Therapeutic Measure, with Notes of Cases Treated.*

BY S. S. WALLIHAN, M. D., CHICAGO.

The relations of oxygen to animal life were early the subject of earnest inquiry.

The experiments of Priestley and Lavoisier demonstrated that birds, rabbits and other animals immersed in an atmosphere of pure oxygen gas were rendered more lively—as it were, stimulated, and that dogs trained for the chase, when allowed to breathe the gas for a short time, could endure greater hardships, and for a longer period, than their fellows who had not been subjected to its influence; and that animals suffocated from inhaling poisonous vapors were speedily resuscitated by a supply of pure oxygen.

It was also demonstrated that the muscles of the animals repeatedly subjected to the influence of the gas, became very firm—toughened as it were, materially interfering with their quality of tenderness for dietetic purposes.

These striking results, naturally, attracted the attention of physiologists, and pointed to important therapeutic developments in the future. Considerable experimentation followed, with a view to make non-atmospheric oxygen available to the intelligent pathologist, in the removal of morbid conditions.

Dr. Beddoes—not "Eddoes" as quoted in the *London Lancet*—has the honor of having been the first medical man who gave any considerable attention to the subject. He was assisted by Sir Humphrey Davy; but great difficulty was experienced in adapting apparatus for the ready evolution of the gas in sufficiently pure condition for medical uses; and after long and painful effort, Beddoes declared himself beaten, not by any lack or loss of faith in oxygen, but "by the difficulties in making it available." His partial success, however, was sufficiently suggestive to inspire further efforts, and he was followed by Drs. Hill, Thornton, Cavallo and others.

Since the former ones were, by the general profession, considered a failure, the experiments of these late investigations were conducted in comparative seclusion, or were made the subject of uncharitable professional criticism and opposition.

Hence, Dr. Hill's work, published in 1820, although full of instructive and striking facts, attracted little notice and was soon forgotten. Few members of the profession, at the present day, are aware that experiments so conclusive were ever conducted, or that a work of such unusual candor and singular ability was ever issued.

Somewhat later, Thornton, Cavallo and McCormack instituted still more extensive trials of the agent, and each unhesitatingly commended it. Within fifteen years past, Richardson, Demarquay, Birch, Goolden, Francis, Alexander, Erichsen and others, have, more or less used, and without exception, added their testimony in approval of the treatment.

In this country, but few members of the profession, of any prominence, have given the subject attention, and the opinion still generally prevails that the inhalation of artificial oxygen, as a therapeutic measure, is of doubtful utility, and, in any event, difficult of accomplishment in general practice. This is scarcely to be wondered at, in view of the fact that literature on the subject is not only meagre but very unsatisfactory. Comparatively nothing on the subject has appeared in this country, and, as yet, no American publisher has had the courage to venture the republication of foreign works of the kind. The medical press generally, has ignored all mention of the subject; the notable exceptions including the *London Lancet*, and *British and Foreign Medico-Chirurgical Review*, in which some interesting cases, treated by means of super-oxygenation, have been reported from time to time, and articles on the same subject have occasionally appeared.

Our own Prof. Cohen, of Jefferson Medical College, in his recent work (*On Inhalation*, Phila. 1867), asserts with great positiveness, the value of pure oxygen in certain forms of disease in which he has used it or witnessed its use. Dr. DaCosta, in his monograph on the same subject, makes cautious mention of it as the "most promising" of all the gases, but dismisses the question of its use by saying, "we must wait the results of these later experiments, since the former ones were unsatisfactory."

Demarquay's reports to the Academy of Medicine, and *Gazette Médicale* (Paris, 1866), are quite conclusive and satisfactory, but have not yet been republished in English. The monograph of Dr. S. B. Birch, of Manchester Medical School, (Second edition, London, 1868.) is probably the most satisfactory contribution yet made to medical literature, on the subject of pure oxygen as a curative agent. The first edition of the same work, issued in 1857, was not well received by the English medical press, but was made the subject of considerable ungenerous criticism, and never found its way to this side.

This author, whose experience with oxygen, extending through a period of more than fifteen years, probably exceeds that of any other practitioner who has yet spoken—asserts, as his conclusions concerning the agent, that, "it is a powerful, really scientific and agreeable curative agent; is capable of far more extensive range in its application to the rational treatment of chronic diseases than, perhaps, any other remedy—*is permanently Nature's own therapeutic*, affording assistance in her own way, without opposing the intentions of her ever-present *vis medicatrix*, and is entitled to the position of a *curative*, in a variety of intractable diseases, otherwise incurable by any other known means, or tacitly acknowledged to be so; * * * is occasionally *the* remedy, and then the only one worthy of the name, in certain contingencies, where life must be (and frequently is) sacrificed by neglecting a fair trial of it." (*On Oxygen, pp. 148*). He adds: "It may with safety be predicted that, sooner or later, non-atmospheric oxygen will be universally admitted as one of our most valued remedial agents. (Op. cit., p. 149.)

It would seem a notable lack of professional acumen or professional enterprise, that so little is known concerning the therapeutic use of this agent, and that so potent an ally in the treatment of disease should not long since have been made more generally available. But the difficulties of rendering it so in general practice have thus far, no doubt, deterred many from attempting its use, who otherwise would be glad to avail themselves of its aid. The impetus of late given to the subject, both in Europe and this country, bids fair to enlist the attention of the general profession, and to secure for the treatment, recognition adequate to its importance.

Of the various methods of exhibiting the remedy, which have been brought forward from time to time, the more practicable and important are,

1. *Direct inhalation of the gas into the lungs*, which is, *par excellence*, at the head of all other methods of administration. The gas may be generated by any of the processes known to chemists, care being taken to secure a sufficient degree of purity.

The process by means of chlorate of potash and per-oxide of manganese, is, undoubtedly, the most practical and economical for ordinary purposes. It requires some sacrifice of time and care in its manipulation, and is, so far, objectionable to the busy practitioner; but the gas afforded by this process can be made of such absolute purity, and the apparatus required is so comparatively inexpensive, that it is to be preferred to any other.

A process for the evolution of the gas in a tolerable state of purity, by means of chloride of lime and per-oxide of cobalt, has lately been perfected by the Messrs. Robbins & Co., of London. It was originally proposed by Flitmann, but was not looked upon with much favor, since it required that the chloride should be in saturated solution previous to the addition of the cobalt, which seriously interferes with the portability of the material and its adaptability to emergencies. By some process of their own, which they very ungraciously decline to impart to any American chemist, the Messrs. R. & Co. manage to combine these substances, in right proportions, in the form of dry powder, which may be preserved any length of time, unchanged, and is ready for instant use on the addition of simple boiling water.

The per-oxide of hydrogen, in solution, raised to a temperature of nearly 100°, gives off limited quantities of perfectly pure oxygen; but this process is expensive and will be brought into requisition only when limited quantities of the gas are required, or when other methods are not at hand.

Per-oxide of barium and bi-chromate of potash, with diluted sulphuric acid, is another process for obtaining the gas, but scarcely to be recommended.

The decomposition of water by the galvanic current may, in time, become a more feasible and less expensive method than now.

(To be continued.)

